# Supplementary material for: Pyruvate Dehydrogenase Kinase 1 inhibition mediated oxidative phosphorylation enhancement in cartilage promotes osteoarthritis progression
Source: BMC Musculoskelet Disord. 2023 Jul 20;24:597. doi: 10.1186/s12891-023-06585-6 (PMC10357736; doi:10.1186/s12891-023-06585-6)
Supplement: Supplementary file 1 — Additional file 1. [file 12891_2023_6585_MOESM1_ESM.zip › KEGG.Gsea.1653622432464/KEGG_ENDOCYTOSIS.html]

Details for gene set KEGG\_ENDOCYTOSIS[GSEA]

|  || Dataset | 55457\_collapsed\_to\_symbols.cls.cls #OA\_versus\_normal.cls.cls #OA\_versus\_normal\_repos |
| Phenotype | cls.cls#OA\_versus\_normal\_repos |
| Upregulated in class | normal |
| GeneSet | KEGG\_ENDOCYTOSIS |
| Enrichment Score (ES) | -0.24839473 |
| Normalized Enrichment Score (NES) | -1.1520377 |
| Nominal p-value | 0.17872968 |
| FDR q-value | 0.44476813 |
| FWER p-Value | 1.0 |
Table: GSEA Results Summary

  

Fig 1: Enrichment plot: KEGG\_ENDOCYTOSIS      
 Profile of the Running ES Score & Positions of GeneSet Members on the Rank Ordered List

  

| SYMBOL | TITLE | RANK IN GENE LIST | RANK METRIC SCORE | RUNNING ES | CORE ENRICHMENT || 1 | RAB4A | "RAB4A, member RAS oncogene family [Source:HGNC Symbol;Acc:HGNC:9781]" | 66 | 0.962 | 0.0127 | No |
| 2 | CHMP2B | charged multivesicular body protein 2B [Source:HGNC Symbol;Acc:HGNC:24537] | 129 | 0.876 | 0.0241 | No |
| 3 | TSG101 | tumor susceptibility 101 [Source:HGNC Symbol;Acc:HGNC:15971] | 284 | 0.763 | 0.0261 | No |
| 4 | LDLRAP1 | low density lipoprotein receptor adaptor protein 1 [Source:HGNC Symbol;Acc:HGNC:18640] | 445 | 0.684 | 0.0263 | No |
| 5 | CHMP4A | charged multivesicular body protein 4A [Source:HGNC Symbol;Acc:HGNC:20274] | 526 | 0.649 | 0.0321 | No |
| 6 | CHMP5 | charged multivesicular body protein 5 [Source:HGNC Symbol;Acc:HGNC:26942] | 556 | 0.638 | 0.0416 | No |
| 7 | AP2S1 | adaptor related protein complex 2 subunit sigma 1 [Source:HGNC Symbol;Acc:HGNC:565] | 577 | 0.630 | 0.0518 | No |
| 8 | ADRB2 | adrenoceptor beta 2 [Source:HGNC Symbol;Acc:HGNC:286] | 596 | 0.625 | 0.0619 | No |
| 9 | HSPA2 | heat shock protein family A (Hsp70) member 2 [Source:HGNC Symbol;Acc:HGNC:5235] | 662 | 0.603 | 0.0680 | No |
| 10 | CSF1R | colony stimulating factor 1 receptor [Source:HGNC Symbol;Acc:HGNC:2433] | 713 | 0.590 | 0.0751 | No |
| 11 | HSPA1L | heat shock protein family A (Hsp70) member 1 like [Source:HGNC Symbol;Acc:HGNC:5234] | 1139 | 0.488 | 0.0508 | No |
| 12 | SH3GLB1 | "SH3 domain containing GRB2 like, endophilin B1 [Source:HGNC Symbol;Acc:HGNC:10833]" | 1216 | 0.473 | 0.0536 | No |
| 13 | STAMBP | STAM binding protein [Source:HGNC Symbol;Acc:HGNC:16950] | 1227 | 0.471 | 0.0615 | No |
| 14 | SMAP1 | small ArfGAP 1 [Source:HGNC Symbol;Acc:HGNC:19651] | 1243 | 0.468 | 0.0690 | No |
| 15 | CHMP2A | charged multivesicular body protein 2A [Source:HGNC Symbol;Acc:HGNC:30216] | 1269 | 0.464 | 0.0757 | No |
| 16 | PIP5K1B | phosphatidylinositol-4-phosphate 5-kinase type 1 beta [Source:HGNC Symbol;Acc:HGNC:8995] | 1291 | 0.459 | 0.0826 | No |
| 17 | AP2M1 | adaptor related protein complex 2 subunit mu 1 [Source:HGNC Symbol;Acc:HGNC:564] | 1419 | 0.441 | 0.0808 | No |
| 18 | RAB11A | "RAB11A, member RAS oncogene family [Source:HGNC Symbol;Acc:HGNC:9760]" | 1447 | 0.436 | 0.0868 | No |
| 19 | ASAP3 | "ArfGAP with SH3 domain, ankyrin repeat and PH domain 3 [Source:HGNC Symbol;Acc:HGNC:14987]" | 1480 | 0.432 | 0.0923 | No |
| 20 | VPS28 | VPS28 subunit of ESCRT-I [Source:HGNC Symbol;Acc:HGNC:18178] | 1522 | 0.426 | 0.0970 | No |
| 21 | VPS45 | vacuolar protein sorting 45 homolog [Source:HGNC Symbol;Acc:HGNC:14579] | 1533 | 0.423 | 0.1040 | No |
| 22 | DAB2 | DAB adaptor protein 2 [Source:HGNC Symbol;Acc:HGNC:2662] | 1821 | 0.381 | 0.0886 | No |
| 23 | ARRB1 | arrestin beta 1 [Source:HGNC Symbol;Acc:HGNC:711] | 2116 | 0.348 | 0.0720 | No |
| 24 | KIT | "KIT proto-oncogene, receptor tyrosine kinase [Source:HGNC Symbol;Acc:HGNC:6342]" | 2117 | 0.348 | 0.0784 | No |
| 25 | GRK6 | G protein-coupled receptor kinase 6 [Source:HGNC Symbol;Acc:HGNC:4545] | 2130 | 0.347 | 0.0839 | No |
| 26 | CHMP6 | charged multivesicular body protein 6 [Source:HGNC Symbol;Acc:HGNC:25675] | 2140 | 0.345 | 0.0896 | No |
| 27 | RAB31 | "RAB31, member RAS oncogene family [Source:HGNC Symbol;Acc:HGNC:9771]" | 2232 | 0.335 | 0.0887 | No |
| 28 | EHD4 | EH domain containing 4 [Source:HGNC Symbol;Acc:HGNC:3245] | 2313 | 0.325 | 0.0884 | No |
| 29 | EGF | epidermal growth factor [Source:HGNC Symbol;Acc:HGNC:3229] | 2399 | 0.315 | 0.0876 | No |
| 30 | ARFGAP2 | ADP ribosylation factor GTPase activating protein 2 [Source:HGNC Symbol;Acc:HGNC:13504] | 2548 | 0.297 | 0.0815 | No |
| 31 | AP2A2 | adaptor related protein complex 2 subunit alpha 2 [Source:HGNC Symbol;Acc:HGNC:562] | 2574 | 0.294 | 0.0850 | No |
| 32 | CCR5 | C-C motif chemokine receptor 5 [Source:HGNC Symbol;Acc:HGNC:1606] | 2617 | 0.289 | 0.0871 | No |
| 33 | CHMP3 | charged multivesicular body protein 3 [Source:HGNC Symbol;Acc:HGNC:29865] | 2733 | 0.277 | 0.0832 | No |
| 34 | PRKCZ | protein kinase C zeta [Source:HGNC Symbol;Acc:HGNC:9412] | 2760 | 0.274 | 0.0862 | No |
| 35 | VPS4B | vacuolar protein sorting 4 homolog B [Source:HGNC Symbol;Acc:HGNC:10895] | 2783 | 0.271 | 0.0895 | No |
| 36 | FGFR3 | fibroblast growth factor receptor 3 [Source:HGNC Symbol;Acc:HGNC:3690] | 2812 | 0.269 | 0.0923 | No |
| 37 | IQSEC1 | IQ motif and Sec7 domain ArfGEF 1 [Source:HGNC Symbol;Acc:HGNC:29112] | 2814 | 0.269 | 0.0972 | No |
| 38 | PDGFRA | platelet derived growth factor receptor alpha [Source:HGNC Symbol;Acc:HGNC:8803] | 2943 | 0.256 | 0.0919 | No |
| 39 | RNF41 | ring finger protein 41 [Source:HGNC Symbol;Acc:HGNC:18401] | 3005 | 0.249 | 0.0918 | No |
| 40 | NTRK1 | neurotrophic receptor tyrosine kinase 1 [Source:HGNC Symbol;Acc:HGNC:8031] | 3015 | 0.248 | 0.0957 | No |
| 41 | SNF8 | SNF8 subunit of ESCRT-II [Source:HGNC Symbol;Acc:HGNC:17028] | 3037 | 0.246 | 0.0986 | No |
| 42 | GRK4 | G protein-coupled receptor kinase 4 [Source:HGNC Symbol;Acc:HGNC:4543] | 3127 | 0.237 | 0.0960 | No |
| 43 | CLTB | clathrin light chain B [Source:HGNC Symbol;Acc:HGNC:2091] | 3131 | 0.237 | 0.1002 | No |
| 44 | WWP1 | WW domain containing E3 ubiquitin protein ligase 1 [Source:HGNC Symbol;Acc:HGNC:17004] | 3230 | 0.227 | 0.0967 | No |
| 45 | SH3GL3 | "SH3 domain containing GRB2 like 3, endophilin A3 [Source:HGNC Symbol;Acc:HGNC:10832]" | 3360 | 0.216 | 0.0906 | No |
| 46 | PIP4K2B | phosphatidylinositol-5-phosphate 4-kinase type 2 beta [Source:HGNC Symbol;Acc:HGNC:8998] | 3416 | 0.210 | 0.0902 | No |
| 47 | RAB5C | "RAB5C, member RAS oncogene family [Source:HGNC Symbol;Acc:HGNC:9785]" | 3433 | 0.208 | 0.0928 | No |
| 48 | CLTCL1 | clathrin heavy chain like 1 [Source:HGNC Symbol;Acc:HGNC:2093] | 3631 | 0.191 | 0.0808 | No |
| 49 | DNAJC6 | DnaJ heat shock protein family (Hsp40) member C6 [Source:HGNC Symbol;Acc:HGNC:15469] | 3665 | 0.189 | 0.0818 | No |
| 50 | PSD3 | pleckstrin and Sec7 domain containing 3 [Source:HGNC Symbol;Acc:HGNC:19093] | 3672 | 0.188 | 0.0848 | No |
| 51 | NEDD4L | NEDD4 like E3 ubiquitin protein ligase [Source:HGNC Symbol;Acc:HGNC:7728] | 3733 | 0.183 | 0.0835 | No |
| 52 | GRK3 | G protein-coupled receptor kinase 3 [Source:HGNC Symbol;Acc:HGNC:290] | 4208 | 0.137 | 0.0488 | No |
| 53 | IL2RA | interleukin 2 receptor subunit alpha [Source:HGNC Symbol;Acc:HGNC:6008] | 4292 | 0.129 | 0.0447 | No |
| 54 | CLTC | clathrin heavy chain [Source:HGNC Symbol;Acc:HGNC:2092] | 4330 | 0.126 | 0.0441 | No |
| 55 | EHD3 | EH domain containing 3 [Source:HGNC Symbol;Acc:HGNC:3244] | 4395 | 0.121 | 0.0413 | No |
| 56 | RAB22A | "RAB22A, member RAS oncogene family [Source:HGNC Symbol;Acc:HGNC:9764]" | 4678 | 0.098 | 0.0210 | No |
| 57 | CLTA | clathrin light chain A [Source:HGNC Symbol;Acc:HGNC:2090] | 4718 | 0.094 | 0.0197 | No |
| 58 | ARAP1 | "ArfGAP with RhoGAP domain, ankyrin repeat and PH domain 1 [Source:HGNC Symbol;Acc:HGNC:16925]" | 4730 | 0.093 | 0.0206 | No |
| 59 | PSD4 | pleckstrin and Sec7 domain containing 4 [Source:HGNC Symbol;Acc:HGNC:19096] | 4749 | 0.091 | 0.0208 | No |
| 60 | EHD2 | EH domain containing 2 [Source:HGNC Symbol;Acc:HGNC:3243] | 4828 | 0.085 | 0.0163 | No |
| 61 | ERBB3 | erb-b2 receptor tyrosine kinase 3 [Source:HGNC Symbol;Acc:HGNC:3431] | 4863 | 0.083 | 0.0152 | No |
| 62 | IL2RG | interleukin 2 receptor subunit gamma [Source:HGNC Symbol;Acc:HGNC:6010] | 4887 | 0.081 | 0.0148 | No |
| 63 | PDCD6IP | programmed cell death 6 interacting protein [Source:HGNC Symbol;Acc:HGNC:8766] | 4956 | 0.076 | 0.0109 | No |
| 64 | IL2RB | interleukin 2 receptor subunit beta [Source:HGNC Symbol;Acc:HGNC:6009] | 5045 | 0.069 | 0.0053 | No |
| 65 | VPS37C | VPS37C subunit of ESCRT-I [Source:HGNC Symbol;Acc:HGNC:26097] | 5060 | 0.068 | 0.0055 | No |
| 66 | ACAP1 | "ArfGAP with coiled-coil, ankyrin repeat and PH domains 1 [Source:HGNC Symbol;Acc:HGNC:16467]" | 5210 | 0.056 | -0.0052 | No |
| 67 | FGFR4 | fibroblast growth factor receptor 4 [Source:HGNC Symbol;Acc:HGNC:3691] | 5351 | 0.044 | -0.0154 | No |
| 68 | RAB11FIP2 | RAB11 family interacting protein 2 [Source:HGNC Symbol;Acc:HGNC:29152] | 5388 | 0.041 | -0.0174 | No |
| 69 | ASAP2 | "ArfGAP with SH3 domain, ankyrin repeat and PH domain 2 [Source:HGNC Symbol;Acc:HGNC:2721]" | 5742 | 0.012 | -0.0449 | No |
| 70 | TRAF6 | TNF receptor associated factor 6 [Source:HGNC Symbol;Acc:HGNC:12036] | 5761 | 0.011 | -0.0461 | No |
| 71 | SH3GL2 | "SH3 domain containing GRB2 like 2, endophilin A1 [Source:HGNC Symbol;Acc:HGNC:10831]" | 6007 | -0.005 | -0.0653 | No |
| 72 | DNM3 | dynamin 3 [Source:HGNC Symbol;Acc:HGNC:29125] | 6020 | -0.006 | -0.0661 | No |
| 73 | GRK5 | G protein-coupled receptor kinase 5 [Source:HGNC Symbol;Acc:HGNC:4544] | 6209 | -0.022 | -0.0805 | No |
| 74 | RAB5B | "RAB5B, member RAS oncogene family [Source:HGNC Symbol;Acc:HGNC:9784]" | 6375 | -0.033 | -0.0928 | No |
| 75 | CXCR4 | C-X-C motif chemokine receptor 4 [Source:HGNC Symbol;Acc:HGNC:2561] | 6382 | -0.034 | -0.0927 | No |
| 76 | STAM | signal transducing adaptor molecule [Source:HGNC Symbol;Acc:HGNC:11357] | 6403 | -0.035 | -0.0936 | No |
| 77 | PIP5K1C | phosphatidylinositol-4-phosphate 5-kinase type 1 gamma [Source:HGNC Symbol;Acc:HGNC:8996] | 6601 | -0.050 | -0.1081 | No |
| 78 | PARD6B | par-6 family cell polarity regulator beta [Source:HGNC Symbol;Acc:HGNC:16245] | 6688 | -0.055 | -0.1138 | No |
| 79 | DNM1 | dynamin 1 [Source:HGNC Symbol;Acc:HGNC:2972] | 6691 | -0.055 | -0.1130 | No |
| 80 | ERBB4 | erb-b2 receptor tyrosine kinase 4 [Source:HGNC Symbol;Acc:HGNC:3432] | 6830 | -0.065 | -0.1226 | No |
| 81 | EHD1 | EH domain containing 1 [Source:HGNC Symbol;Acc:HGNC:3242] | 6846 | -0.067 | -0.1225 | No |
| 82 | SRC | "SRC proto-oncogene, non-receptor tyrosine kinase [Source:HGNC Symbol;Acc:HGNC:11283]" | 6940 | -0.075 | -0.1284 | No |
| 83 | ARAP2 | "ArfGAP with RhoGAP domain, ankyrin repeat and PH domain 2 [Source:HGNC Symbol;Acc:HGNC:16924]" | 6983 | -0.079 | -0.1303 | No |
| 84 | AGAP1 | "ArfGAP with GTPase domain, ankyrin repeat and PH domain 1 [Source:HGNC Symbol;Acc:HGNC:16922]" | 7081 | -0.086 | -0.1363 | No |
| 85 | RUFY1 | RUN and FYVE domain containing 1 [Source:HGNC Symbol;Acc:HGNC:19760] | 7125 | -0.090 | -0.1380 | No |
| 86 | HLA-F | "major histocompatibility complex, class I, F [Source:HGNC Symbol;Acc:HGNC:4963]" | 7196 | -0.096 | -0.1417 | No |
| 87 | RAB11B | "RAB11B, member RAS oncogene family [Source:HGNC Symbol;Acc:HGNC:9761]" | 7227 | -0.097 | -0.1423 | No |
| 88 | ARRB2 | arrestin beta 2 [Source:HGNC Symbol;Acc:HGNC:712] | 7255 | -0.100 | -0.1425 | No |
| 89 | MDM2 | MDM2 proto-oncogene [Source:HGNC Symbol;Acc:HGNC:6973] | 7273 | -0.101 | -0.1420 | No |
| 90 | MET | "MET proto-oncogene, receptor tyrosine kinase [Source:HGNC Symbol;Acc:HGNC:7029]" | 7322 | -0.104 | -0.1438 | No |
| 91 | CHMP1B | charged multivesicular body protein 1B [Source:HGNC Symbol;Acc:HGNC:24287] | 7339 | -0.105 | -0.1431 | No |
| 92 | FGFR2 | fibroblast growth factor receptor 2 [Source:HGNC Symbol;Acc:HGNC:3689] | 7374 | -0.108 | -0.1438 | No |
| 93 | NEDD4 | NEDD4 E3 ubiquitin protein ligase [Source:HGNC Symbol;Acc:HGNC:7727] | 7407 | -0.111 | -0.1443 | No |
| 94 | AGAP2 | "ArfGAP with GTPase domain, ankyrin repeat and PH domain 2 [Source:HGNC Symbol;Acc:HGNC:16921]" | 7732 | -0.136 | -0.1672 | No |
| 95 | HSPA1A | heat shock protein family A (Hsp70) member 1A [Source:HGNC Symbol;Acc:HGNC:5232] | 7876 | -0.146 | -0.1757 | No |
| 96 | HRAS | "HRas proto-oncogene, GTPase [Source:HGNC Symbol;Acc:HGNC:5173]" | 7977 | -0.153 | -0.1807 | No |
| 97 | USP8 | ubiquitin specific peptidase 8 [Source:HGNC Symbol;Acc:HGNC:12631] | 8031 | -0.158 | -0.1819 | No |
| 98 | RAB5A | "RAB5A, member RAS oncogene family [Source:HGNC Symbol;Acc:HGNC:9783]" | 8049 | -0.159 | -0.1803 | No |
| 99 | RABEP1 | "rabaptin, RAB GTPase binding effector protein 1 [Source:HGNC Symbol;Acc:HGNC:17677]" | 8165 | -0.168 | -0.1862 | No |
| 100 | ADRB1 | adrenoceptor beta 1 [Source:HGNC Symbol;Acc:HGNC:285] | 8212 | -0.172 | -0.1866 | No |
| 101 | SH3GL1 | "SH3 domain containing GRB2 like 1, endophilin A2 [Source:HGNC Symbol;Acc:HGNC:10830]" | 8440 | -0.189 | -0.2009 | No |
| 102 | F2R | coagulation factor II thrombin receptor [Source:HGNC Symbol;Acc:HGNC:3537] | 8447 | -0.190 | -0.1979 | No |
| 103 | GRK2 | G protein-coupled receptor kinase 2 [Source:HGNC Symbol;Acc:HGNC:289] | 8470 | -0.191 | -0.1960 | No |
| 104 | DNM1L | dynamin 1 like [Source:HGNC Symbol;Acc:HGNC:2973] | 8499 | -0.194 | -0.1946 | No |
| 105 | HSPA8 | heat shock protein family A (Hsp70) member 8 [Source:HGNC Symbol;Acc:HGNC:5241] | 9140 | -0.243 | -0.2404 | No |
| 106 | GIT1 | GIT ArfGAP 1 [Source:HGNC Symbol;Acc:HGNC:4272] | 9236 | -0.251 | -0.2432 | No |
| 107 | RET | ret proto-oncogene [Source:HGNC Symbol;Acc:HGNC:9967] | 9297 | -0.256 | -0.2431 | No |
| 108 | PLD1 | phospholipase D1 [Source:HGNC Symbol;Acc:HGNC:9067] | 9365 | -0.261 | -0.2435 | Yes |
| 109 | EPS15 | epidermal growth factor receptor pathway substrate 15 [Source:HGNC Symbol;Acc:HGNC:3419] | 9409 | -0.265 | -0.2420 | Yes |
| 110 | CBL | Cbl proto-oncogene [Source:HGNC Symbol;Acc:HGNC:1541] | 9479 | -0.272 | -0.2424 | Yes |
| 111 | GIT2 | GIT ArfGAP 2 [Source:HGNC Symbol;Acc:HGNC:4273] | 9552 | -0.278 | -0.2429 | Yes |
| 112 | CBLC | Cbl proto-oncogene C [Source:HGNC Symbol;Acc:HGNC:15961] | 9553 | -0.279 | -0.2377 | Yes |
| 113 | PLD2 | phospholipase D2 [Source:HGNC Symbol;Acc:HGNC:9068] | 9615 | -0.284 | -0.2372 | Yes |
| 114 | CXCR2 | C-X-C motif chemokine receptor 2 [Source:HGNC Symbol;Acc:HGNC:6027] | 9666 | -0.289 | -0.2358 | Yes |
| 115 | PARD6A | par-6 family cell polarity regulator alpha [Source:HGNC Symbol;Acc:HGNC:15943] | 9703 | -0.292 | -0.2332 | Yes |
| 116 | MVB12B | multivesicular body subunit 12B [Source:HGNC Symbol;Acc:HGNC:23368] | 9842 | -0.305 | -0.2383 | Yes |
| 117 | PIP5K1A | phosphatidylinositol-4-phosphate 5-kinase type 1 alpha [Source:HGNC Symbol;Acc:HGNC:8994] | 9851 | -0.306 | -0.2333 | Yes |
| 118 | CXCR1 | C-X-C motif chemokine receptor 1 [Source:HGNC Symbol;Acc:HGNC:6026] | 9936 | -0.315 | -0.2340 | Yes |
| 119 | RAB11FIP3 | RAB11 family interacting protein 3 [Source:HGNC Symbol;Acc:HGNC:17224] | 9961 | -0.317 | -0.2300 | Yes |
| 120 | AP2B1 | adaptor related protein complex 2 subunit beta 1 [Source:HGNC Symbol;Acc:HGNC:563] | 10119 | -0.333 | -0.2362 | Yes |
| 121 | TFRC | transferrin receptor [Source:HGNC Symbol;Acc:HGNC:11763] | 10184 | -0.339 | -0.2349 | Yes |
| 122 | ASAP1 | "ArfGAP with SH3 domain, ankyrin repeat and PH domain 1 [Source:HGNC Symbol;Acc:HGNC:2720]" | 10276 | -0.349 | -0.2356 | Yes |
| 123 | HGS | hepatocyte growth factor-regulated tyrosine kinase substrate [Source:HGNC Symbol;Acc:HGNC:4897] | 10315 | -0.352 | -0.2320 | Yes |
| 124 | ARFGAP3 | ADP ribosylation factor GTPase activating protein 3 [Source:HGNC Symbol;Acc:HGNC:661] | 10382 | -0.359 | -0.2305 | Yes |
| 125 | EPN2 | epsin 2 [Source:HGNC Symbol;Acc:HGNC:18639] | 10446 | -0.367 | -0.2286 | Yes |
| 126 | RAB11FIP5 | RAB11 family interacting protein 5 [Source:HGNC Symbol;Acc:HGNC:24845] | 10457 | -0.369 | -0.2226 | Yes |
| 127 | HSPA6 | heat shock protein family A (Hsp70) member 6 [Source:HGNC Symbol;Acc:HGNC:5239] | 10561 | -0.379 | -0.2236 | Yes |
| 128 | SH3GLB2 | "SH3 domain containing GRB2 like, endophilin B2 [Source:HGNC Symbol;Acc:HGNC:10834]" | 10566 | -0.380 | -0.2169 | Yes |
| 129 | IQSEC3 | IQ motif and Sec7 domain ArfGEF 3 [Source:HGNC Symbol;Acc:HGNC:29193] | 10573 | -0.381 | -0.2103 | Yes |
| 130 | RAB11FIP1 | RAB11 family interacting protein 1 [Source:HGNC Symbol;Acc:HGNC:30265] | 10710 | -0.394 | -0.2136 | Yes |
| 131 | VPS4A | vacuolar protein sorting 4 homolog A [Source:HGNC Symbol;Acc:HGNC:13488] | 10768 | -0.401 | -0.2107 | Yes |
| 132 | FLT1 | fms related receptor tyrosine kinase 1 [Source:HGNC Symbol;Acc:HGNC:3763] | 10826 | -0.408 | -0.2076 | Yes |
| 133 | ARFGAP1 | ADP ribosylation factor GTPase activating protein 1 [Source:HGNC Symbol;Acc:HGNC:15852] | 10827 | -0.408 | -0.2000 | Yes |
| 134 | HLA-G | "major histocompatibility complex, class I, G [Source:HGNC Symbol;Acc:HGNC:4964]" | 10869 | -0.413 | -0.1956 | Yes |
| 135 | ARF6 | ADP ribosylation factor 6 [Source:HGNC Symbol;Acc:HGNC:659] | 11084 | -0.442 | -0.2041 | Yes |
| 136 | EEA1 | early endosome antigen 1 [Source:HGNC Symbol;Acc:HGNC:3185] | 11118 | -0.445 | -0.1985 | Yes |
| 137 | STAM2 | signal transducing adaptor molecule 2 [Source:HGNC Symbol;Acc:HGNC:11358] | 11194 | -0.456 | -0.1959 | Yes |
| 138 | ACAP2 | "ArfGAP with coiled-coil, ankyrin repeat and PH domains 2 [Source:HGNC Symbol;Acc:HGNC:16469]" | 11229 | -0.461 | -0.1900 | Yes |
| 139 | KDR | kinase insert domain receptor [Source:HGNC Symbol;Acc:HGNC:6307] | 11454 | -0.496 | -0.1984 | Yes |
| 140 | IQSEC2 | IQ motif and Sec7 domain ArfGEF 2 [Source:HGNC Symbol;Acc:HGNC:29059] | 11494 | -0.500 | -0.1921 | Yes |
| 141 | PSD | pleckstrin and Sec7 domain containing [Source:HGNC Symbol;Acc:HGNC:9507] | 11597 | -0.515 | -0.1906 | Yes |
| 142 | HLA-E | "major histocompatibility complex, class I, E [Source:HGNC Symbol;Acc:HGNC:4962]" | 11659 | -0.526 | -0.1856 | Yes |
| 143 | ADRB3 | adrenoceptor beta 3 [Source:HGNC Symbol;Acc:HGNC:288] | 11691 | -0.531 | -0.1782 | Yes |
| 144 | EPN3 | epsin 3 [Source:HGNC Symbol;Acc:HGNC:18235] | 11749 | -0.541 | -0.1726 | Yes |
| 145 | ITCH | itchy E3 ubiquitin protein ligase [Source:HGNC Symbol;Acc:HGNC:13890] | 11800 | -0.549 | -0.1663 | Yes |
| 146 | SMURF2 | SMAD specific E3 ubiquitin protein ligase 2 [Source:HGNC Symbol;Acc:HGNC:16809] | 11802 | -0.550 | -0.1562 | Yes |
| 147 | VPS37B | VPS37B subunit of ESCRT-I [Source:HGNC Symbol;Acc:HGNC:25754] | 11894 | -0.570 | -0.1528 | Yes |
| 148 | LDLR | low density lipoprotein receptor [Source:HGNC Symbol;Acc:HGNC:6547] | 12009 | -0.595 | -0.1507 | Yes |
| 149 | EGFR | epidermal growth factor receptor [Source:HGNC Symbol;Acc:HGNC:3236] | 12109 | -0.622 | -0.1469 | Yes |
| 150 | HSPA1B | heat shock protein family A (Hsp70) member 1B [Source:HGNC Symbol;Acc:HGNC:5233] | 12278 | -0.670 | -0.1476 | Yes |
| 151 | SMURF1 | SMAD specific E3 ubiquitin protein ligase 1 [Source:HGNC Symbol;Acc:HGNC:16807] | 12344 | -0.688 | -0.1399 | Yes |
| 152 | PIKFYVE | "phosphoinositide kinase, FYVE-type zinc finger containing [Source:HGNC Symbol;Acc:HGNC:23785]" | 12347 | -0.690 | -0.1273 | Yes |
| 153 | DNM2 | dynamin 2 [Source:HGNC Symbol;Acc:HGNC:2974] | 12362 | -0.695 | -0.1155 | Yes |
| 154 | ARAP3 | "ArfGAP with RhoGAP domain, ankyrin repeat and PH domain 3 [Source:HGNC Symbol;Acc:HGNC:24097]" | 12386 | -0.704 | -0.1042 | Yes |
| 155 | PRKCI | protein kinase C iota [Source:HGNC Symbol;Acc:HGNC:9404] | 12444 | -0.723 | -0.0953 | Yes |
| 156 | PARD3 | par-3 family cell polarity regulator [Source:HGNC Symbol;Acc:HGNC:16051] | 12489 | -0.743 | -0.0849 | Yes |
| 157 | HLA-A | "major histocompatibility complex, class I, A [Source:HGNC Symbol;Acc:HGNC:4931]" | 12502 | -0.749 | -0.0720 | Yes |
| 158 | HLA-B | "major histocompatibility complex, class I, B [Source:HGNC Symbol;Acc:HGNC:4932]" | 12534 | -0.764 | -0.0602 | Yes |
| 159 | CDC42 | cell division cycle 42 [Source:HGNC Symbol;Acc:HGNC:1736] | 12681 | -0.852 | -0.0559 | Yes |
| 160 | IGF1R | insulin like growth factor 1 receptor [Source:HGNC Symbol;Acc:HGNC:5465] | 12689 | -0.855 | -0.0405 | Yes |
| 161 | CBLB | Cbl proto-oncogene B [Source:HGNC Symbol;Acc:HGNC:1542] | 12725 | -0.887 | -0.0268 | Yes |
| 162 | EPN1 | epsin 1 [Source:HGNC Symbol;Acc:HGNC:21604] | 12833 | -1.037 | -0.0160 | Yes |
| 163 | GRK1 | G protein-coupled receptor kinase 1 [Source:HGNC Symbol;Acc:HGNC:10013] | 12874 | -1.139 | 0.0020 | Yes |
Table: GSEA details [plain text format]

  

Fig 2: KEGG\_ENDOCYTOSIS      
 Blue-Pink O' Gram in the Space of the Analyzed GeneSet

  

Fig 3: KEGG\_ENDOCYTOSIS: Random ES distribution      
 Gene set null distribution of ES for **KEGG\_ENDOCYTOSIS**
